# Association of oxidative stress, programmed cell death, GSTM1 gene polymorphisms, smoking and the risk of lung carcinogenesis: A two-step Mendelian randomization study

**DOI:** 10.3389/fphys.2023.1145129

**Published:** 2023-04-18

**Authors:** Yijun Wang, Qiongling Yang, Lingzhen Zheng

**Affiliations:** Zhangzhou Affiliated Hospital of Fujian Medical University, Zhangzhou, China

**Keywords:** oxidative stress, programmed cell death, GSTM1 gene polymorphisms, smoking, lung carcinogenesis

## Abstract

**Aim:** We aimed to examine the association of oxidative stress, programmed cell death, smoking, and the *GSTM1* gene in the risk of lung carcinogenesis. The two-step Mendelian randomization will reveal evidence supporting the association of the exposure and mediators with the resulting outcome.

**Methods:** In step 1, we estimated the impact of smoking exposure on lung carcinogenesis and programmed cell death. Our study involved a total of 500,000 patients of European ancestry, from whom we obtained genotype imputation information. Specifically, we genotyped two arrays: the UK Biobank Axiom (UKBB) which accounted for 95% of marker content, and the UK BiLIEVE Axiom (UKBL). This allowed us to unmask the association between smoking exposure and the incidence of lung carcinogenesis. In step 2, we further examined the effects of smoking on oxidative stress, programmed cell death, and the incidence of lung carcinogenesis.

**Results:** Different outcomes emerged from the two-step Mendelian randomization. The GSTM1 gene variant was found to be critical in the development of lung carcinogenesis, as its deletion or deficiency can induce the condition. A GWAS study on participant information obtained from the UK Biobank revealed that smoking interferes with the GSTM1 gene, causing programmed cell death in the lungs and ultimately leading to lung carcinogenesis. The relative risk of developing lung carcinogenesis associated with oxidative stress was significantly high among current smokers (a hazard ratio of 17.8, 95% confidence interval of 12.2–26.0) and heavy smokers (a hazard ratio of 16.6 and a 95% confidence interval of 13.6–20.3) compared to individuals who never smoked. The GSTM1 gene polymorphism was found to be 0.006 among participants who have never smoked, <0.001 among ever-smokers, and 0.002 and <0.001 among current and former smokers, respectively.

We compared the effect of smoking within two particular time frames, 6 years and 55 years, and found that smoking’s impact on the GSTM1 gene was highest among participants who were 55 years old. The genetic risk peaked among individuals aged 50 years and above (PRS of at least 80%).

**Conclusion:** Exposure to smoking is a significant factor in developing lung carcinogenesis, as it is associated with programmed cell death and other mediators involved in the condition. Oxidative stress caused by smoking is also a key mechanism in lung carcinogenesis. The results of the present study highlight the association between oxidative stress, programmed cell death, and the GSTM1 gene in the development of lung carcinogenesis.

## 1 Introduction

Lung cancer is primarily caused by smoking, with developing countries accounting for 70% of all related deaths. The global prevalence of lung cancer is approximately 42 million (Adibhesami et al., 2018). The World Health Organization reports that smoking is the leading cause of lung cancer and contributes to up to 25% of cancer-related deaths (World Health Organization, 2019). Smokers are 22 times more likely to develop lung cancer than non-smokers, and 40% of tobacco-related deaths result from lung diseases, including cancer (Garwood, 2019). Epidemiological data and statistics support this close association between smoking and lung cancer, and a potent causal-effect relationship exists.

Mendelian randomization (MR) is a statistical technique used to examine causal inferences in observational studies (Zhang et al., 2022). It uses single-nucleotide polymorphisms from genome-wide association studies (GWAS) to analyze the association with an exposure variable (Zhang et al., 2022). The Glutathione S Transferase mu 1 (GSTM1) gene has been linked with lung cancer risk, as it plays a role in toxicity and detoxification (Zhang et al., 2022). Deleting this gene has increased the risk of lung cancer (Yu et al., 2018). Thus, smoking and GSTM1 play significant health roles. Previous studies have reported that GSTM1 deficiency can potentially increase the adverse effects of exposure to smoke (Yang et al., 2015).

Chemicals in tobacco produce free radicals in the body, which cause oxidative stress that weakens the host’s defense system. Oxidative stress is associated with lung carcinogenesis (Kabesch et al., 2004). Low concentrations of reactive oxygen species (ROS) generated by oxidative stress are involved in developing and progressing lung tumors, especially non-small cell lung cancer (NSCLC). High concentrations of ROS can initiate the autophagic apoptotic progression of tumor cells and promote the sensitivity of NSCLC cells to chemoradiotherapy. Programmed cell death, which refers to an active demise process that occurs to maintain homeostasis after a cell receives a specific signal or is stimulated by certain factors, is also closely related to the course of lung cancer. Cigarette smoking promotes programmed cell death (Reuter et al., 2010).

Previous studies have reported a high correlation between GSTM1 and lung carcinogenesis, with evidence suggesting that individuals with common GSTM1 polymorphisms, including other Glutathione S-transferase genes, could be more susceptible to lung cancer upon exposure to tobacco (Arfin, 2021). In this study, we examined the role of GSTM1 polymorphisms in lung carcinogenesis and the association between smoking and oxidative stress and programmed cell death. Our findings suggest that exposure to smoking is associated with lung carcinogenesis, while oxidative stress and programmed cell death are thought to be associated with smoking (and therefore strongly associated with the disease). We also studied the role of the gene variant in the development of lung disease, alongside its possible influence on programmed cell death and oxidative stress.

## 2 Methods

### 2.1 Exposure and outcome data sources

We used a population of at least 500,000 adults from three countries: Scotland, England, and Wales obtained from the UK Biobank. The inclusion criteria involved patients aged 40 years and above, patients diagnosed with lung cancer, and patients with a history of smoking tobacco or data related to their daily, weekly, or monthly smoking schedules. Basic demographic features were collected through a computer-assisted and touch-screen questionnaire. Diagnosis and data on the incidence of lung carcinogenesis were obtained from the National Health Service Information Center for patients in Wales and England and the NHS Central Register Scotland for Scottish patients, with a follow-up up to March 2016 and October 2015, respectively. Lung carcinogenesis was coded according to the International Classification of Diseases, 10th Revision (ICD-10) or the International Classification of Diseases, Ninth Revision (ICD-9), and patients were identified with codes 34 and 162.2 to 162.9 in each case.

We obtained genotype imputation information from 488,377 patients from the UK Biobank by genotyping two arrays, namely, the UK Biobank Axiom (UKBB), which accounted for 95% of marker content, and the UK BiLIEVE Axiom (UKBL). The genotype data was inputted through the Haplotype Reference Consortium’s reference panels with the UK10K haplotype resources. To ensure the quality of the data, we applied exclusion criteria to patients with other cancers or chronic lung infections, those who had been exposed to chemotherapy or various radiations, and those who did not consent to participate in the study. We excluded 628 patients, who were identified as outliers for sex chromosome aneuploidy and heterozygosity.

We identified European patients displaying genotype information of all samples in the first main components for the four 1,000 genome populations, namely, JPT, YRI, CEU, and CHB. We excluded 23,425 patients as they did not fall within the CEU cluster. Additionally, we excluded 90,924 participants below the age of 50 years, patients diagnosed with cancer at baseline (n = 24,944), second and higher-degree relatives (n = 37,590 determined using KING-robust kinship estimator ≥0.0422), and patients with unknown smoking values (n = 1,398). After applying the exclusion criteria, we were left with 308,490 patients for the investigation, comprising 164,327 females and 144,173 males.

Smoking was considered the exposure of interest, while genetic variants were instrumental variables. In addition, smoking (as an environmental factor) and GSTM1 polymorphisms were regarded as mediators of the association between programmed cell death and oxidative stress.

### 2.2 Mendelian randomization methods and polygenic risk score building

To investigate the causal relationship between modifiable exposures such as smoking and the risk of developing lung cancer, we utilized a dual-step sample Mendelian randomization approach. In this approach, we used smoking-related genetic variants as instrumental variables. We identified a total of 32 loci associated with lung cancer in previous studies with a *p*-value <5.0 × 10^−8^, out of which 18 were excluded from the present investigation due to being excluded in previous studies, having minor allele frequencies less than 0.01, or being in linkage disequilibrium with other variants (r2 > 0.2) (Bhara et al., 2019). This left us with 19 variants from 14 loci for constructing a polygenic risk score (PRS) for lung carcinogenesis. Supplementary Table S1 lists these 19 single-nucleotide polymorphisms. We obtained variant-specific weights by utilizing regression coefficients from genome-wide studies involving 56,450 controls and 29,266 cases of European ancestry (Bossé and Amos, 2017). The PRS was calculated as the sum of the product of the number and weight of risk alleles in each risk variant per individual. A list of the risk variants used for constructing the PRS of lung cancer can be found in Supplementary Tables S2–S4.

### 2.3 Survival analysis

We analyzed the progression of the primary lung cancers—squamous cell carcinoma, adenocarcinoma, and non-small cell lung cancer, where possible interactions of every risk variant or PRS (with smoking) were assessed through likelihood test ratios. We used the chi-square and Mann-Whitney-Wilcoxon tests for categorical baseline variables and univariate analyses (Bhara et al., 2019). We relied on the proportional hazard models to estimate 95% Confidence Intervals (95% CIs) and hazard ratios (HRs), where age was set as a time scale truncated at enrollment, of lung carcinoma linked with smoking and PRS via quantiles. Individuals who did not report pack-per-year data were separately grouped.

We proceeded by estimating the risk of lung carcinoma for patient groups defined by PRS and smoking status: contrasting heavy smokers and never smokers. The covariates in this analysis include age, education (secondary education, university degree or college), sex, genotype array (UKBB or UKBL), and ancestry’s main components. We used the Schoenfeld residuals to check the assumptions on proportional hazards. In contrast, the Cox regression technique was used to estimate the absolute risk of lung carcinogenesis for 6 years for patients in a group cumulatively characterized by smoking status and levels of PRS, where a threshold of 0.0151 was applied as the threshold for eligibility screening. We adjusted according to the mode of categorical covariates and the mean of continuous covariates in the model.

We derived this threshold value from a significant study reporting outcomes from a low-dose computed tomography and a chest X-ray, where mortality was 6 years, significantly decreasing the risk of lung carcinogenesis at 0.0151 or above. The significance level of the two-sided tests was maintained at a *p* = 0.05. Lastly, we visualized the graphs using the R programming language. We fed the raw data into the program and drew the plots concerning the outcomes of the present study.

### 2.4 Ethical approval

We did not require ethical approval for this study as it involved collecting and analyzing published documents and existing data previously approved by the ethics committee.

## 3 Results

During the median follow-up period, there were 1,449 incidences of lung carcinogenesis observed over a maximum of 5.8 years. A comparison between non-cases and cases is presented in Table 1. Cases were more likely to be smokers with a higher number of pack-years, older, male, and have lower levels of education compared to non-cases.

**TABLE 1:** Basic characteristics of the included participants for lung carcinogenesis: non-cases and cases in the UK Biobank. We examined six basic characteristics of participants included in the study for lung carcinogenesis: age, sex, educational qualifications, smoking status (never smokers, former smokers, and current smokers), pack-years for smokers, and PRS for lung cancer. The table below shows a comparison of non-cases and cases. Cases are likely to be older, male, have lower educational qualifications, and be smokers with more pack-years of smoke than non-cases. A *p*-value of less than 0.05 was considered statistically significant for all characteristics examined in cases and non-cases.

Characteristics	Cases (n = 1449)	Non-cases (n = 307,041)	*p*-value
Age, mean (SD)	62.5 (4.8)	60.0 (5.4)	<.001
Sex, no. (%)			<.001
**Female**	662 (45.7)	163,665 (53.3)	
**Male**	787 (54.3)	143,386 (46.7)	
Education, no (%)			<.001
**College or university degree**	232 (16)	97,266 (31.7)	
**Some professional qualifications**	364 (25.1)	82,897 (27.0)	
**Secondary education**	259 (17.9)	65,102 (21.2)	
**None of the above**	594 (41.0)	61,776 (20.1)	
Smoking Status			<.001
**Never smokers**	179 (12.4)	160,908 (52.4)	
**Former smokers**	668 (46.1)	117,416 (38.2)	
**Current smokers**	602 (41.5)	28,717 (9.35)	
Pack-years for smokers, mean (SD)a	42.1 (25.2)	25.0 (19.6)	<.001
PRS for lung cancer, mean (SD)	1.97 (0.4)	1.90 (0.40)	<.001

Out of 211,180 participants, 161,087 reported that they had never smoked. However, 50,093 participants who did not report pack years were excluded from the study.

Table 2 presents the statistical outcomes of the association between smoking status and lung carcinogenesis. The results show that compared to non-smokers, smoking individuals are more susceptible to lung carcinogenesis. Heavy smokers (those who smoked at least 30 packs per year) had a hazard ratio of 19.9 (95% confidence interval of 16.8–23.6) compared to individuals who had never smoked. Similarly, smokers who smoked 20–29 packs of cigarettes were also more susceptible to lung carcinogenesis than non-smokers (hazard ratio of 20.7%, 95% confidence interval of 16.3–26.4). We found that heavy smokers were more susceptible to lung carcinogenesis than other groups of smokers, especially former smokers who had smoked 20–29 packs of cigarettes. However, those who quit smoking within the last 15 years were less susceptible to lung carcinogenesis (hazard ratio of 7.45%, 95% confidence interval of 5.37–10.3) than heavy smokers who had quit at least 15 years ago (hazard ratio of 14.8%, 95% confidence interval of 12.2–17.8). Similar trends were observed in the progression of other types of lung cancers.

**TABLE 2:** The hazard ratios (95% CI) for lung carcinoma were associated with smoking status in the UK Biobank study. The study classified smoking status as never, heavy, current, and former smokers. Former smokers were categorized based on quitting time, which was determined to be about 15 years, and pack years ranged from 20 to 29.

Smoking status	No. Of cases	HR (95% CI)^a^
Never Smokers	179	1 (reference)
Heavy Smokers^b^	654	19.9 (16.8–23.6)
**Current Smokers**	365	27.6 (23.0–33.1)
Former smokers, quit-time≤15 years	289	14.8 (12.2–17.8)
Other current smokers		
**20–29 pack-years**	106	20.7 (16.3–26.4)
**< 20 pack-years**	58	9.83 (7.30–13.2)
Missing pack-year information	73	8.88 (6.75–11.7)
Other former smokers		
**≥ 30 pack-years, quit-time > 15 years**	63	6.29 (4.70–8.42)
**20–29 pack-years, quit-time≤15 years**	45	7.45 (5.37–10.3)
**20–29 pack-years, quit-time > 15 years**	50	4.23 (3.08–5.79)
**< 20 pack-years, quit-time≤15 years**	18	3.40 (2.10–5.53)
**< 20 pack-years, quit-time > 15 years**	80	1.92 (1.47–2.50)
Missing pack-year information	123	2.38 (1.89–3.00)

According to Table 2.

^a^ Hazard ratios were adjusted for education and sex.

^b^ Individuals with a smoking history of at least 30 packs per year and currently smoked or quit within the last 15 years.

161, 087 participants had never smoked, whereas 50,093 participants who did not report pack-years were excluded from the study.

We investigated the association between the risk of lung carcinogenesis and PRS percentile groups and found a statistically significant association between PIR and lung carcinogenesis. This association was observed across every smoking status, and the dose-response outcomes are presented in Table 3 and Table 4. We observed similar patterns in non-small cell lung cancers and adenocarcinoma. We evaluated interaction tests between PRS and smoking status using the multiplicative scale but did not find statistically significant outcomes (interaction *p*-values were>0.05). However, we found a statistically significant interaction between the two variants with a *p*-value <0.05. However, we did not observe a statistically significant association upon adjusting the comparisons.

**TABLE 3:** The hazard ratios (95% confidence interval) for the association of lung carcinogenesis and smoking were analyzed using the UK Biobank data. The PRS was compared among five percentile groups, Q1 (lowest), Q2, Q3, Q4, and Q5 (highest), along with changes in their *p*-values. A statistically significant *p*-value was considered less than 0.05.

PRS	PRS	PRS	PRS	PRS
Q1 (Lowest)	1 (Reference)	1 (Reference)	1 (Reference)	1 (Reference)
Q2	1.12 (0.67–1.89)	1.18 (0.95–1.45)	1.32 (0.98–1.77)	1.04 (0.77–1.41)
Q3	1.24 (0.74–2.06)	1.34 (1.01–1.52)	1.17 (0.87–1.58)	1.31 (0.98–1.74)
Q4	1.37 (0.83–2.24)	1.42 (1.16–1.73)	1.44 (1.08–1.92)	1.40 (1.06–1.85)
Q5 (Highest)	1.93 (1.21–3.07)	1.57 (1.29–1.91)	1.55 (1.17–2.06)	1.58 (1.21–2.08)
*p*-value	0.003	<0.001	0.002	<0.001

According to Table 3.

^a^ The hazard ratios were adjusted for education, sex, genotype array, the first ten primary components, quit time for smokers only, and pack-years for smokers. The interaction between ever smokers and never smokers was not statistically significant (*p* = 0.185), and there was also no significant association between current smokers, never smokers, and former smokers (*p* = 0.778).

**TABLE 4:** Approximated risks of lung carcinogenesis were analyzed in four groups characterized by joint PRS quantiles and smoking status in the UK Biobank. Smoking status was defined by never smokers, current heavy smokers, former heavy smokers, current less heavy smokers, other current smokers, and other former smokers. The smoking status groups were compared from Q1 (lowest) to Q5 (highest) based on hazard ratios and the number of cases.

Q1 lowest		Q2		Q3		Q4		Q5 (Highest)	
No. Cases	HR^a^	No. Cases	HR^a^	No. Cases	HR^a^	No. Cases	HR^a^	No. Cases	HR^a^
27	1 (Reference)	30	1.11 (0.66–1.87)	33	1.23 (0.74–2.05)	37	1.36 (0.83–2.24)	52	1.90 (1.20–3.03)
51	25.4 (15.9–40.6)	75	36.1 (23.2–56.1)	73	32.7 (21.0–51.0)	84	36.9 (23.9–57.1)	82	36.1 (23.3–55.9)
45	15.1 (9.37–24.4)	49	15.5 (9.67–24.8)	55	17.6 (11.1–27.9)	63	19.1 (12.2–30.1)	77	22.4 (14.4–34.8)
17	19.4 (10.5–35.6)	17	19.8 (10.8–36.4)	15	18.0 (9.57–33.9)	21	27.0 (15.2–47.8)	36	45.5 (27.6–75.0)
16	6.92 (3.73–12.9)	21	9.51 (5.37–16.8)	30	14.3 (8.48–24.0)	33	15.8 (9.50–26.3)	31	16.1 (9.62–27.0)
56	2.88 (1.82–4.55)	67	3.47 (2.22–5.43)	87	4.51 (2.93–6.95)	82	4.33 (2.80–6.69)	87	4.63 (3.00–7.13)

According to Table 4.

^a^ Adjusted hazard ratios for education, sex, genotype array, and the first ten important components.

^b^ Current heavy smokers with a smoking history of at least 30 pack-years.

^c^ Former heavy smokers with a history of at least 30 pack-years, but quit in the 15 years.

^d^ Current mild smokers with a history of 20–29 pack-years.

We jointly classified smoking status and PRS levels and tabulated the results in Table 4. When compared to never smokers and those with low risks of lung carcinogenesis based on the lowest PRS quantile, current smokers with 20–29 pack-years and the highest PRS quantile had a hazard ratio of 45.5 (95% confidence interval of 27.6–75.0), indicating a higher risk of lung carcinogenesis. Similarly, heavy smokers with the highest risk of lung carcinogenesis had a hazard ratio of 36.1 (95% confidence interval of 23.2–55.9).

**FIGURE 1:**
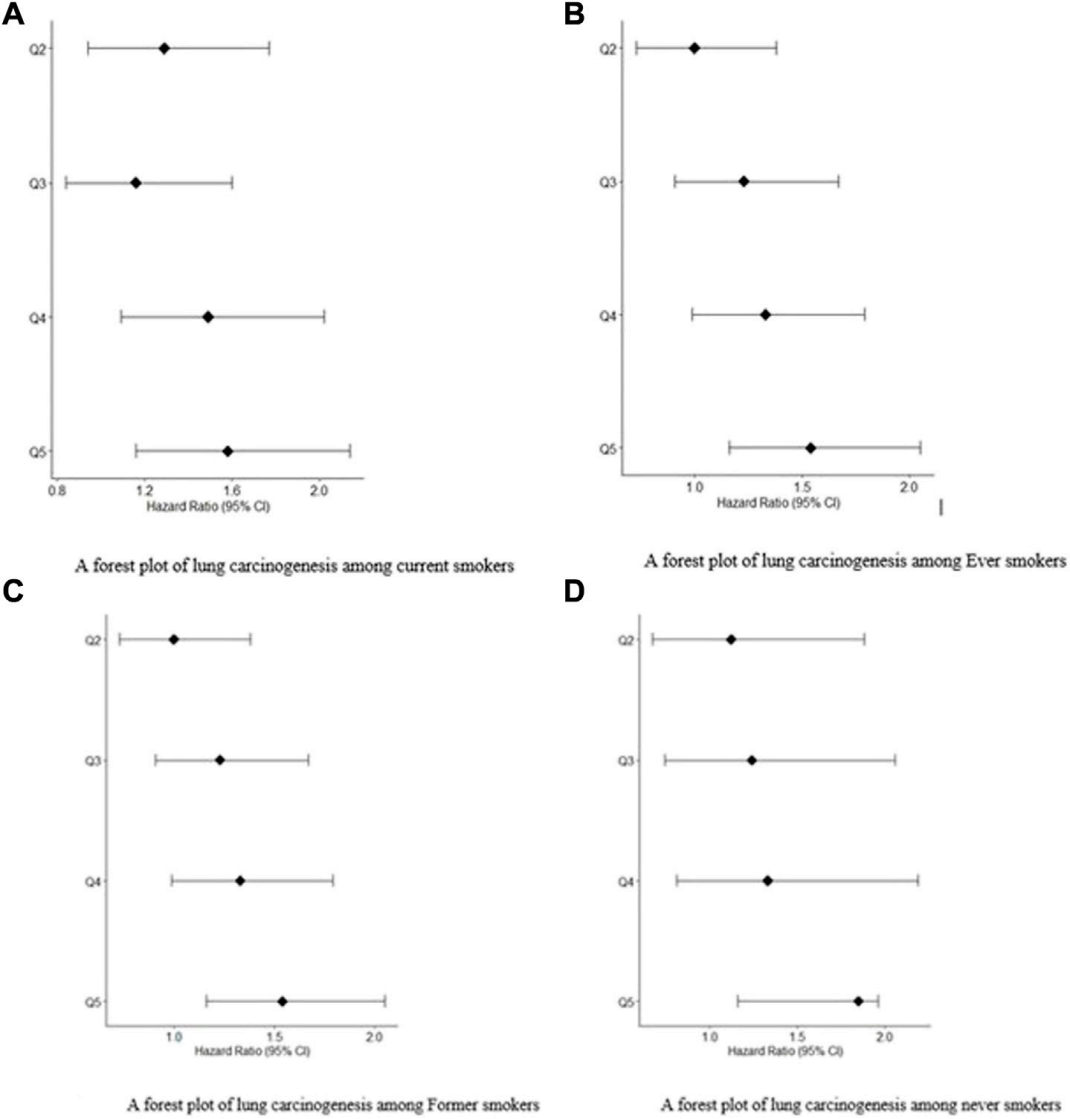
**(A)** A forest plot of lung carcinogenesis among current smokers. **(B)** A forest plot of lung carcinogenesis among Ever smokers. **(C)** A forest plot of lung carcinogenesis among Former smokers. **(D)** A forest plot of lung carcinogenesis among never smokers.

We analyzed data from the UK Biobank to estimate the 6-year risk of lung carcinogenesis and visualized it based on PRS for individuals who never smoked, ever smokers, current smokers, and former smokers (Figure 1). The group of individuals who never smoked had the lowest incidence of lung carcinogenesis (176). In contrast, incidences of lung carcinogenesis for ever-smokers, current smokers, and former smokers were relatively high at 934, 449, and 485, respectively. We analyzed the GSTM1 polymorphism gene trend and found statistically significant values of 0.006 for never-smokers, <0.001 for ever-smokers, and 0.002 and <0.001 for current and former smokers, respectively.

We compared the effect of smoking within particular time frames (6 years and 55 years) and found that the effect of smoking on the GSTM1 gene was at its maximum among participants who were 55 years or older. The genetic risk was highest among participants aged 50 years or older, with a PRS of at least 80%

The prevalence of lung carcinogenesis among individuals who never smoked, ever smokers, current smokers, and former smokers. Figures 1A,B are forest plots for patients exposed to.

Smoking: current smokers and ever smokers, respectively. Figures 1C,D are forest plots for patients exposed to smoking: former smokers and never smokers, respectively. In all the forest plot, Q5 had the greatest hazard ratios at 95% confidence Intervals compared to Q4, Q3 and Q2.

**FIGURE 2:**
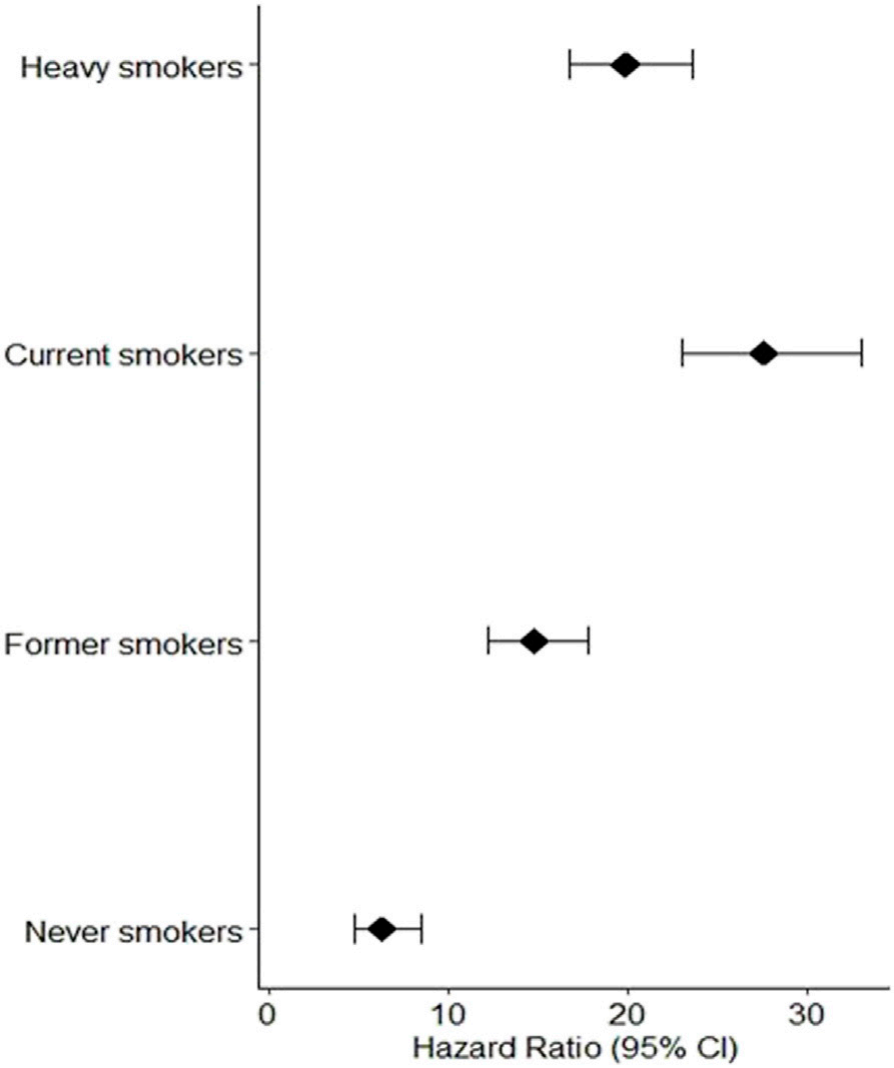
Individuals who had never smoked had lower levels of programmed cell death than the other groups, including ever.

We investigated the relationship between lung carcinogenesis, GSTM1 genes, smoking, and programmed cell death using data from the UK Biobank. Our analysis revealed that individuals who had never smoked had lower levels of programmed cell death than the other groups, including ever-smokers, current smokers, and former smokers. The results are shown in Figure 2 below.

The hazard ratios (95% CI) for the four groups of participants showed that current heavy smokers had the greatest risk of developing lung cancer, followed by current less heavy smokers, former smokers, and individuals who never smoked. As shown in Figure 2, the observed low programmed cell death among individuals who never smoked can be attributed to the absence of smoking exposure, a major risk factor for lung cancer due to carcinogenic chemicals in tobacco smoke. This absence of smoking exposure may result in lower levels of oxidative stress and programmed cell death, contributing to the lower risk of lung cancer among never smokers compared to smokers.

The mediator of GSTM1 gene mutation and deletion was also considered. These genetic variants can affect the detoxification of carcinogenic compounds in tobacco smoke, leading to increased levels of oxidative stress and DNA damage, which can result in programmed cell death or cell survival depending on the severity and duration of the oxidative stress. Therefore, GSTM1 gene polymorphisms could potentially mediate the effect of smoking on oxidative stress and programmed cell death.

**FIGURE 3:**
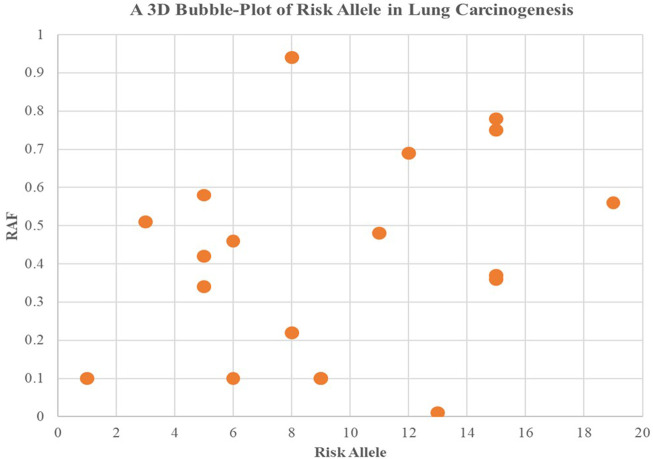
We created a bubble plot to display the risk alleles associated with lung carcinogenesis. The *y*-axis indicates the risk allele frequency, while the *x*-axis represents the risk allele’s association with developing lung cancer. Each point on the plot corresponds to specific alleles at varying frequencies, and the size of the bubbles corresponds to the effect size of their corresponding risk allele.

**FIGURE 4:**
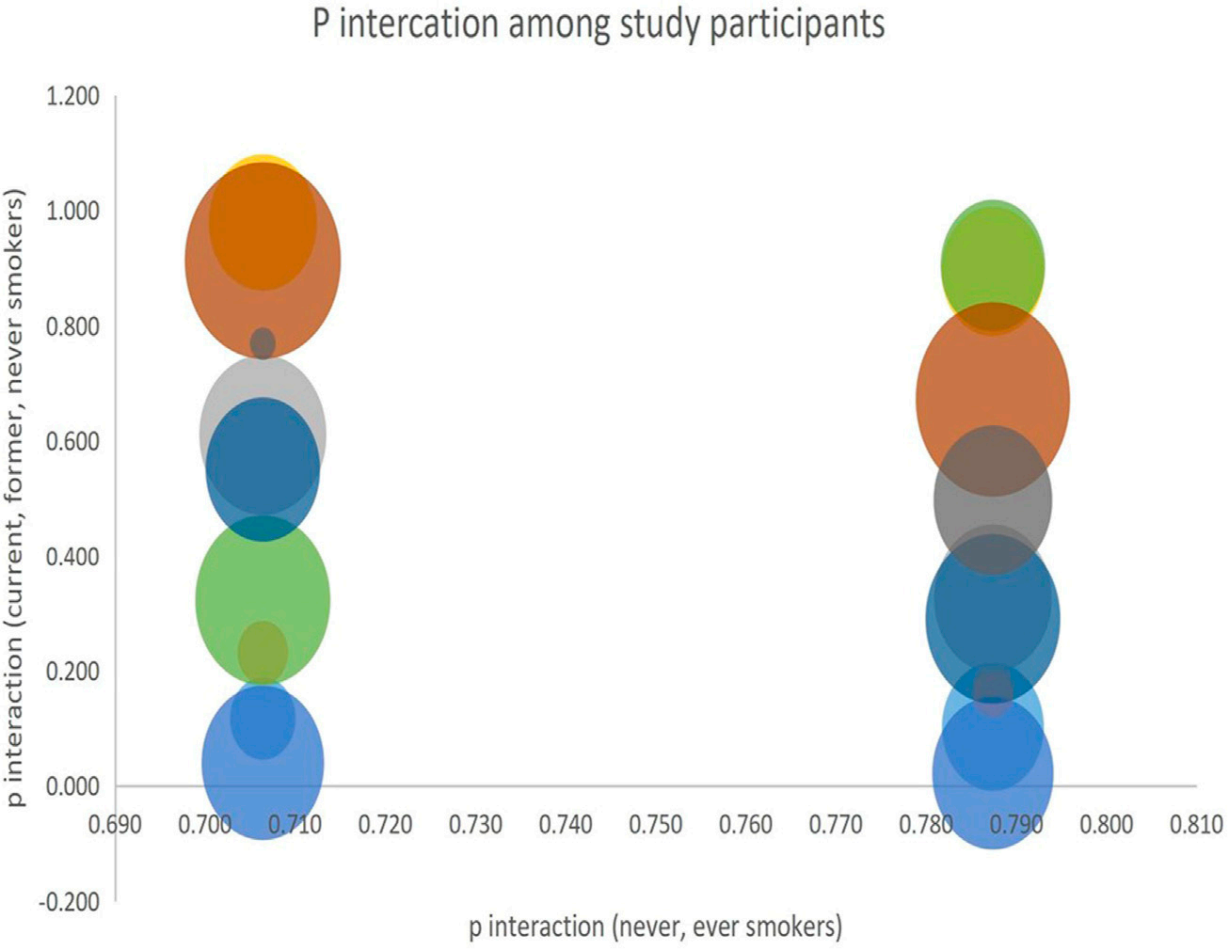
P interaction among current, former, and never smokers vs-a-vis never, ever smokers.

Higher levels of programmed cell death were observed in the groups with high incidence rates of lung carcinogenesis, as shown in Figure 4. This relationship between smoking and lung carcinogenesis can be attributed to the high levels of programmed cell death resulting from smoking. Indeed, smoking has been found to mediate programmed cell death. To further illustrate the role of risk alleles in lung carcinogenesis, we created a bubble plot using data obtained from the GWAS database. (Figure 3).

We analyzed the interaction of the alleles among the four study groups using *p*-values obtained from the subgroups and visualized the results. P-interactions are statistical techniques used to examine the effect of a variable on an outcome whose effect is regulated by a third variable. In this study, we used p-interaction to examine the effects of smoking on the risk of developing lung cancer in patient groups who were smokers, never smoked, or had a history of smoking. A significant P-interaction implies that smoking has a significant effect in increasing the risk of developing lung cancer. The results of our analysis are presented in Figure 4.

## 4 Discussion

In our Mendelian randomization analysis, we investigated the recently proposed approach by the United States Preventive Service Task Force (USPSTF) to screen and treat lung carcinogenesis in individuals of European ancestry who smoke. Our study provides evidence suggesting that smoking increases the risk of developing lung carcinogenesis. Our findings support the causal role of GSTM1 polymorphisms in mediating the influence of smoking on lung carcinogenesis. Specifically, smoking increased lung carcinogenesis through the GSTM1 gene and oxidative stress, with GSTM1 null genotype carriers having a higher risk of lung carcinogenesis when exposed to smoking.

Moreover, we identified programmed cell death as a mechanism underlying the association between smoking and lung cancer. Our study found that exposure to smoking was associated with programmed cell death in lung tissue, contributing to the development of lung cancer. Thus, our study provides evidence for the causal role of genetic (GSTM1 polymorphisms) and environmental (smoking) factors in lung carcinogenesis, with oxidative stress and programmed cell death as essential mediators of this association.

We also found that the risk of developing lung carcinogenesis was accompanied by programmed cell death, with smokers at the highest risk of programmed cell death in lung tissues. Further investigations revealed that the severity of the disease process was implicated by the number of cigarettes smoked, with the duration of smoking significantly influencing the disease process (Kamceva et al., 2016).

Our analysis of GWAS data revealed that current smokers (20–29 pack-years) and heavy smokers with a history of at least 30 pack-years were equally susceptible to developing lung carcinogenesis. Smoking or a history of smoking was associated with high genetic risks, including deletion and deficiency of the GSTM1 gene, where smokers and heavy smokers were in the highest PRS quantile, reaching the threshold of 50 years of age. These findings are consistent with another study performed in China, where smokers who smoked for approximately 20–29 pack-years were found to have a higher risk of genetic disposition to lung carcinogenesis than non-smokers (up to 5% in PRS), alongside a cumulative risk of lung carcinogenesis among heavy smokers who smoked at least 30 pack-years, with measurements taken at the intermediate risk of genetic deficiency or deletion (McKay et al., 2017).

Our findings align with the theoretical perspectives on smoking, programmed cell death, and the GSTM1 gene’s role in lung carcinogenesis. The GSTM1 gene detoxifies metabolites of environmental carcinogens like tobacco smoke (Kabesch et al., 2004; Reuter et al., 2010; Zhu et al., 2013; Yang et al., 2015; Adibhesami et al., 2018; Yu et al., 2018; Arfin, 2021; Zhang et al., 2022). Its deletion or inadequacy increases susceptibility to lung cancer. Some previous investigations have reported an increased susceptibility to lung carcinogenesis among individuals with common polymorphisms of glutathione S-transferase genes, such as GSTT1 and GSTP1. Other studies have linked polymorphisms in these genes to toxicity, pharmacogenetics, and treatment outcomes. Despite smoking being the most cited risk factor for lung carcinogenesis, genetics also plays a significant role in incidence and progression (Schwartz and Cote, 2015; Cancer Genetics, 2019).

To estimate the risk of genetic factors in lung carcinogenesis, we established a PRS. We avoided PRS overfitting by selecting risk variants identified through previous GWAS studies. We used regression coefficients from the previous GWAS as weights to establish the PRS.

Surprisingly, GWAS and statistical analysis reported a high incidence rate of lung carcinogenesis among non-smokers, which raises questions about the GSTM1 gene polymorphism. Theoretically, the deletion or deficiency of the gene is associated with developing lung carcinogenesis and/or via programmed cell death. An investigation examined the GSTM1 gene’s status among non-smokers to assess possible gene inheritance and its role in lung carcinogenesis. The study found that about 50% of human beings inherit deleted types of the GSTM1 gene (Lam et al., 2019). This phenomenon leaves approximately half of the global population vulnerable to lung carcinogenesis due to a lack of detoxification of carcinogenic substances, as the deletion process causes the GSTM1 gene’s function to be lost. The considerable number of non-smokers found with progressing lung cancer in our study could be attributed to this cause.

Smoking is known to increase oxidative stress (Bell et al., 1993), characterized by increased oxidative damage and decreased antioxidant defense, and is proportional to the number of cigarettes smoked. Our study found a similar risk of lung cancer among current and heavy smokers (with a smoking history of 20–29 pack-years who had quit within the last 15 years). The relative risk of lung cancer associated with oxidative stress was 17.8 (95% confidence interval of 12.2–26.0) among current smokers and 16.6 (95% confidence interval of 13.6–20.3) among heavy smokers compared to individuals who never smoked.

Our cohort consisted of 654 lung cancer patients who met the screening criteria of the USPSTF-2014 guidelines, as well as 106 current smokers with a smoking history of 20–29 pack-years. We found that 16.2% of the study population (106 out of 654 patients) could be screened and diagnosed with lung cancer, per the USPSTF’s recommendation that former smokers with a smoking history of 20–29 pack years who quit within the last 15 years are at a higher risk of oxidative stress, programmed cell death, and GSTM1 deficiency or deletion.

However, further studies are needed to provide robust evidence, as our analysis involved a few study participants (n = 45).

### 4.1 Future and clinical implications

Our study has established the causal role of smoking in lung carcinogenesis and suggests that smoking cessation can prevent lung cancer. This conclusion has significant clinical implications for healthcare providers in educating patients about the risks of smoking and encouraging them to quit smoking. In addition, our study has clinical implications for potential therapeutic targets in treating and diagnosing lung cancer, such as antioxidants. Furthermore, we propose future research to investigate the significance of genetic factors, such as GSMT1 gene polymorphisms, in lung cancer, which can help researchers develop personalized treatments for patients based on their genetic risk profiles.

### 4.2 Limitations

Our study had some limitations. Firstly, the small study population, particularly the non-smokers and current smokers, limited the assessment of lung carcinogenesis risk based on PRS analysis. However, the outcomes were essential for comparing and determining the effect of smoking on lung cancer, considering the modulators and confounding factors. Secondly, the small number of patients diagnosed with lung carcinogenesis at age 50 to 55 (n = 158) limited the observation of a 6-year outcome of lung carcinogenesis risk. Future studies should include larger participant groups in this age range for robust evidence. Finally, the follow-up period was short, and future studies should consider longer follow-up periods for accurate risk calculation.

## 5 Conclusion

Our study has revealed smoking as a key modulator of lung carcinogenesis, mainly through the GSTM1 gene, where its deficiency and deletion were significant factors. Interestingly, we also found a significant prevalence of lung carcinogenesis among non-smokers, albeit less than among those exposed to smoke. Smoking was observed to enhance oxidative stress, and the number of cigarettes smoked by the patients was found to be proportional to the level of carcinogenesis, with long smoking durations carrying a higher probability. Even patients who had quit smoking were found to be vulnerable to carcinogenesis, and both current and heavy smokers were equally susceptible. Age also emerged as a mediator in lung carcinogenesis.

Our study has important clinical implications for public health initiatives aimed at reducing smoking prevalence and promoting smoking cessation, as well as informing clinical guidelines and recommendations for screening, diagnosis, and treatment of lung cancer. By identifying the molecular pathways involved in lung cancer development, our study may also inform the development of new treatments and personalized approaches to lung cancer management.

## Data Availability

The original contributions presented in the study are included in the article/Supplementary Material, further inquiries can be directed to the corresponding author.
