# Supplementary material for: Association of oxidative stress, programmed cell death, GSTM1 gene polymorphisms, smoking and the risk of lung carcinogenesis: A two-step Mendelian randomization study
Source: Front Physiol. 2023 Apr 18;14:1145129. doi: 10.3389/fphys.2023.1145129 (PMC10151499; doi:10.3389/fphys.2023.1145129)
Supplement: Supplementary file 1 [file Table1.DOCX]

| **Smoking status** | **No. of cases** | **HR (95% CI)**a |
| --- | --- | --- |
| Never smokers | 179 | 1 (reference) |
| Heavy smokersb | 654 | 19.9 (16.8–23.6) |
| Current smokers | 365 | 27.6 (23.0–33.1) |
| Former smokers, quit-time ≤ 15 years | 289 | 14.8 (12.2–17.8) |
| Other current smokers |  |  |
| 20–29 pack-years | 106 | 20.7 (16.3–26.4) |
|  | 58 | 9.83 (7.30–13.2) |
| Missing pack-year information | 73 | 8.88 (6.75–11.7) |
| Other former smokers |  |  |
| ≥30 pack-years, quit-time >15 years | 63 | 6.29 (4.70–8.42) |
| 20–29 pack-years, quit-time ≤ 15 years | 45 | 7.45 (5.37–10.3) |
| 20–29 pack-years, quit-time > 15 years | 50 | 4.23 (3.08–5.79) |
|  | 18 | 3.40 (2.10–5.53) |
|  | 80 | 1.92 (1.47–2.50) |
| Missing pack-years information | 123 | 2.38 (1.89–3.00) |
|  |  |  |

Supplementary Table 1
