# Supplementary material for: Association of oxidative stress, programmed cell death, GSTM1 gene polymorphisms, smoking and the risk of lung carcinogenesis: A two-step Mendelian randomization study
Source: Front Physiol. 2023 Apr 18;14:1145129. doi: 10.3389/fphys.2023.1145129 (PMC10151499; doi:10.3389/fphys.2023.1145129)
Supplement: Supplementary file 3 [file Table3.DOCX]

**Supplementary Table 3**

Selected risk variants for the polygenic risk score (PRS) of lung cancer**.]**

**Weight**

| **Locus** | **RSID** | **Chr** | **Position (hg19)** | **Risk allele** | **RAF** | **(beta)** | **P-value** |
| --- | --- | --- | --- | --- | --- | --- | --- |
|  |  |  |  |  | 0.1 |  |  |
| 1p31.1 | rs71658797 | 1 | 77967507 | A | 0 | 0.128 | 3.25E-11 |
|  |  |  |  |  | 0.5 |  |  |
| 3q28 | rs13080835 | 3 | 189357199 | G | 1 | 0.057 | 1.25E-06 |
|  |  |  |  |  | 0.3 |  |  |
| 5p15.33 | rs7705526 | 5 | 1285974 | A | 4 | 0.117 | 1.01E-18 |
|  |  |  |  |  | 0.4 |  |  |
| 5p15.33 | rs2853677 | 5 | 1287194 | G | 2 | 0.111 | 2.66E-18 |
|  |  |  |  |  | 0.5 |  |  |
| 5p15.33 | rs465498 | 5 | 1325803 | A | 8 | 0.141 | 2.68E-32 |
|  |  |  |  |  | 0.1 |  |  |
| 6p21.33 | rs3115672 | 6 | 31727897 | T | 0 | 0.166 | 3.47E-18 |
|  |  |  |  |  | 0.4 |  |  |
| 6q27 | rs6920364 | 6 | 167376466 | C | 6 | 0.068 | 1.29E-08 |
|  |  |  |  |  | 0.2 |  |  |
| 8p12 | rs4236709 | 8 | 32410110 | G | 2 | 0.064 | 5.88E-06 |
|  |  |  |  |  | 0.9 |  |  |
| 8p21.2 | rs11780471 | 8 | 27344719 | G | 4 | 0.141 | 1.69E-08 |
|  |  |  |  |  | 0.1 |  |  |
| 9p21.3 | rs885518 | 9 | 21830157 | G | 0 | 0.088 | 2.13E-06 |
|  |  |  |  |  | 0.1 |  |  |
| 9p21.3 | rs62560775 | 9 | 22052068 | G | 0 | 0.100 | 6.02E-07 |
|  |  |  |  |  | 0.4 |  |  |
| 11q23.3 | rs1056562 | 11 | 118125625 | T | 8 | 0.066 | 1.92E-08 |
|  |  |  |  |  | 0.6 |  |  |
| 12p13.33 | rs7953330 | 12 | 998819 | G | 9 | 0.087 | 6.10E-12 |
| 13q13.1 | rs11571833 | 13 | 32972626 | T | 0.0 | 0.472 | 6.12E-16 |

|  |  |  |  |  | 1 |  |  |
| --- | --- | --- | --- | --- | --- | --- | --- |
|  |  |  |  |  | 0.3 |  |  |
| 15q21.1 | rs66759488 | 15 | 47577451 | A | 6 | 0.068 | 2.83E-08 |
|  |  |  |  |  | 0.7 |  |  |
| 15q21.1 | rs77468143 | 15 | 49376624 | T | 5 | 0.083 | 1.00E-09 |
|  |  |  |  |  | 0.3 |  | 3.10E- |
| 15q25.1 | rs55781567 | 15 | 78857986 | G | 7 | 0.260 | 103 |
|  |  |  |  |  | 0.7 |  |  |
| 15q25.1 | rs8042374 | 15 | 78908032 | A | 8 | 0.232 | 2.91E-60 |
|  |  |  |  |  | 0.5 |  |  |
| 19q13.2 | rs56113850 | 19 | 41353107 | C | 6 | 0.123 | 5.02E-19 |

Note: Weight and p-value were reported from study PMCID: PMC5510465. RAF: risk allele frequency.
